# Evaluating person-centered care in residential care facilities from the perspective of caregivers in South Korea: a survey

**DOI:** 10.1186/s12913-023-09490-9

**Published:** 2023-05-17

**Authors:** Young-Ran Chin, Hyo Young Lee

**Affiliations:** 1 Department of Nursing, Chungwoon University, Chungnam, South Korea; 2 Department of Health Administration, Dongseo University, 47 Jurae-ro, Sasang-gu, Busan, 47011 South Korea

**Keywords:** Residential care facilities, Person-centered care, Assessment questionnaire, Caregivers, South Korea

## Abstract

**Background:**

As many older people spend their time in residential care facilities, the demand for person-centered care (PCC), which affects their quality of life, is increasing. Many residential care facility residents have cognitive problems, such as dementia and strokes. Providing quality care upholds their rights as human beings. Currently, the PCC tools used in South Korea are only translations of foreign tools into Korean, so it is necessary to develop tools for older adult care facilities that reflect the reality of Korean care facilities for older adults. This study aims to develop a tool for measuring PCC in residential care facilities for older people from the perspectives of care givers.

**Methods:**

The draft of 34 questions was developed through literature reviews, interviews with LTC practitioners and researchers. This developed questionnaire was then administered to 402 direct caregivers working in the residential care facilities because many of the residents had cognitive problems. By measuring the interrater reliability, the items with high levels of agreement were selected and the validity of the construct was checked through factor analysis. To determine whether the domains adequately measured each concept, we calculated correlation coefficients and Cronbach’s α.

**Results:**

Four domains and 32 items concerning service conditions, resident’s right to self-determination, a comfortable living environment for all residents, and resident and staff satisfaction are derived, thus explaining 24.7%, 23.6%, 14.6%, and 8.00% of the total variance, respectively. Cronbach’s alphas for each domain are 0.965, 0.948, 0.652, and 0.525, respectively, thus demonstrating internal consistency. The inter-rater agreement is high (66.7%~100.0%). The correlation between service conditions and resident’s right to self-determination (r = 0.643, p < 0.001), a comfortable living environment for all residents, resident and staff satisfaction (r = 0.674, p < 0.001), and resident’s right to self-determination and comfortable living environment (r = 0.695, p < 0.001) is strong.

**Conclusions:**

It is important that caregivers recognize PCC and provide services. When evaluating the residential care services, measuring the degree of PCC should be made compulsory. If the facility becomes more person-centered, it will be possible to promote quality of life for older people.

**Trial registration:**

Not applicable.

## Background

As South Korea’s population is aging at the fastest rate in the world, the long-term care (LTC) insurance for older people was introduced in July 2008 for providing residential facilities and home services to older people, with separate financial resources from the National Health Insurance [1, 2]. The stay of older adults in long-term care facilities is on the rise as the Korean government supports the use of long-term care services for older adults through public social insurance from 2008 [2]. The number of beds in residential LTC facilities of South Korea were 24.8 per 1,000 people, aged 65 and over, in 2019; the number of LTC facility users was 2.7 persons among those aged 65 and over, and 8.7 among people aged 80 and over [1]. For facility admittance, 2.6 persons of those aged 65 and over, and 7.1 persons of people aged 80 and over were admitted to facilities, respectively. When comparing these numerical values to the rates of other countries, the fact that South Korea’s values were low but reported high residence rates of LTC facilities for older adults in South Korea than official residence rates [1, 2]. As in other countries, LTC facilities for older adults in South Korea are for those who have difficulties in daily life due to dementia, cerebrovascular disease, or other chronic diseases.

As older population increases worldwide and the number of older adult people in institutions increases, interest in improving service quality through the PCC model is growing in healthcare and LTC systems [1, 3]. PCC is based on Carl Rogers’ theory and Tom Kitwood applied Carl Rogers’ concept to patients with dementia and emphasized the importance of the environment including caregiver attitudes, communication and care practices [4, 5]. They provided an important theoretical rationale for developing different approaches to dementia care, such as behavior-oriented approaches; emotion-oriented approaches, cognition-oriented approaches, and stimulation-oriented approaches (e.g., recreational therapies and multisensory stimulation) [6]. Meanwhile, Brooker applied the “VIPS” framework for PCC application. The “VIPS” includes the following four components. “V” means “valuing people with dementia;” “I” means “individualized care;” “P” means “understanding the world from the patient’s perspective;” and “S” means “providing a social environment that supports the needs of the patients” [7]. Scholl suggested 6 activities to realize PCC: patient information; patient involvement in care; involvement of family and friends; patient empowerment; physical support and emotional support [8]. Furthermore, McCormack and McCance proposed a “framework for PCC nursing” that broadly extends PCC to the entire area of ​​nursing [9].

PCC describes the conditions required for implementation on three levels (structure, process, and outcome) and the following essential prerequisites: at the organizational level for successful implementation of PCC: creating a PCC culture; development and application of educational programs, including health promotion and prevention programs; personnel equipped with the capability to do PCC; providing a supportive and accommodating PCC environment; developing and integrating structures to support health information technology; and structures to measure and monitor PCC. The framework claims that all systems of an organization (e.g. a nursing home or hospital ward) have to support personhood [3].

The theoretical basis and sub-elements of PCC, the intervention framework and contents are very diverse in each literature, and the interventions using the concept of PCC are very diverse and their evaluation is also diverse [3–11]. However, the common focus of all PCCs is the individual person’s expectations, needs, wishes, and preferences beyond the medical condition [12]. And that includes a holistic approach, with relationships and personal values and beliefs as core elements of care [13–15]. PCC, as a holistic approach, aims to enhance autonomy, choice, sense of personal control, independence, and interactions with other people, is one of the main dementia-specific care approaches [16].

It is a philosophy of care that is built around the needs of the individual and contingent upon understanding each unique individual through an interpersonal relationship. Whether it is referred to as “person-directed,” “resident-focused,” “human-centered,” or something similar, its core principles are essentially the same [4]. Person-centered dementia care had significant effects on decreasing behavioral symptoms and psychotropic medication used for residents with dementia in LTC facilities. Implementing a PCC has a positive impact on staff’s attitudes, beliefs and behaviors towards people at the end of life in any setting, including acute care [17].

The need for PCC was emphasized worldwide and guidelines were presented [18–21]. Based on this, studies applying interventions are being conducted, and they are systematically reviewed to prove their effectiveness [6, 16, 22–24]. However, in South Korea, the concept of PCC has been introduced and tools to measure it have been developed to such an extent that only researchers, not practitioners, are interested in it. The PCC tools used in South Korea [25, 26] are only translations of foreign tools into Korean, so it is necessary to develop tools for older adult care facilities that reflect perspectives of caregivers working in Korean older adult care facilities. The PCC has not received much attention due to provider-oriented organization operations, poor service quality, and indifference from the government and related organizations [27–29]. Physical assault, neglect, various human rights violations, and other abuse against older residents in LTC facilities are occasionally reported [27–29], so a person-centered approach to care is more necessary.

A variety of tools to assess PCC practices can currently be found in the literature [3, 6, 18, 24, 30, 31]. Edvardsson and Innes conducted a critical comparative review of published tools measuring the person-centeredness of care for older people and those with dementia. They recommended that their validity, reliability, and applicability should be further explored [30]. Burke et al. developed the Person-Centered Environment and Care Assessment Tool (PCECAT) to assess and improve residential care standards using person-centered principles, while also meeting Australian care guidelines for older adults [31]. A study on the PCC measurement for LTC facilities is the PCC Assessment Tool [32] and Person-centered Climate Questionnaire-Resident version [33], developed by Edvardsson et al. Its validity is translated into Korean [25], in a study that proved its reliability [26]. A study that developed a PCC measurement tool for daycare centers for older adults was translated into Korean and its reliability and validity was proven [34].

In South Korea, interest in PCC is not high, and the proportion of PCC in official LTC institution evaluations is low [35]. To comply with legal obligations, evaluation of LTC facilities is carried out every three years by the National Health Insurance Corporation, the insurer of LTC insurance for older adults, dividing it into document and on-site evaluations [36]. The evaluation consists of five evaluation areas with 50 indicators. The areas are: institutional operation, environment and safety, protection of beneficiaries’ rights, benefit provision process, and benefit provision results. Among them, the indicators under areas that can be said to be related to PCC are: the guarantee of beneficiaries’ right to know, reinforcement of beneficiary (guardian or family member) participation, family and community connection with residents, and guarantee of dignity and privacy of residents in the guarantee of beneficiary rights’ and protection of human rights for residents; 12 points out of 100 are allocated [35]. The evaluation results are published on the relevant website, so that the public can refer to them when selecting service institutions, and LTC institutions receive incentives or negative incentives according to the evaluation results. Therefore, LTC institutions make efforts to receive a high grade [36].

There are practical concerns about how much it is realistically possible to provide PCC that emphasizes “personal characteristics and needs” in older adult care facilities [3, 28, 29]. It is difficult to use the tools for measuring PCC in residential care facilities accurately, as only 45.5% of LTC residents’ ratings in 2019 in South Korea [36]. In addition, there was quite high convergence between the PCC evaluations from the staff, users, and relatives, with correlations ranging between 0.62 and 0.76 [37, 38]. The impact of PCC approaches gave positive influences on stress, burnout, and job satisfaction of staff caring for people with dementia in residential care communities [6]. If PCC has such a positive effect on the staff, problems caused by frequent staff turnover can be reduced and it will have a positive impact on the recipients. Therefore, it is reasonable to conduct a survey on the LTC facility staff, and this study [32] that measures person-centeredness may have limitations in not reflecting the Korean situation because foreign tools that were developed 10 years ago were adapted and utilized. Therefore, we intend to develop a tool for measuring PCC for the staff of residential care facilities that fully reflects the person-centered concept and reflects the latest practice, and to verify the reliability and validity of the tool.

## Methods

**Fig. 1:**
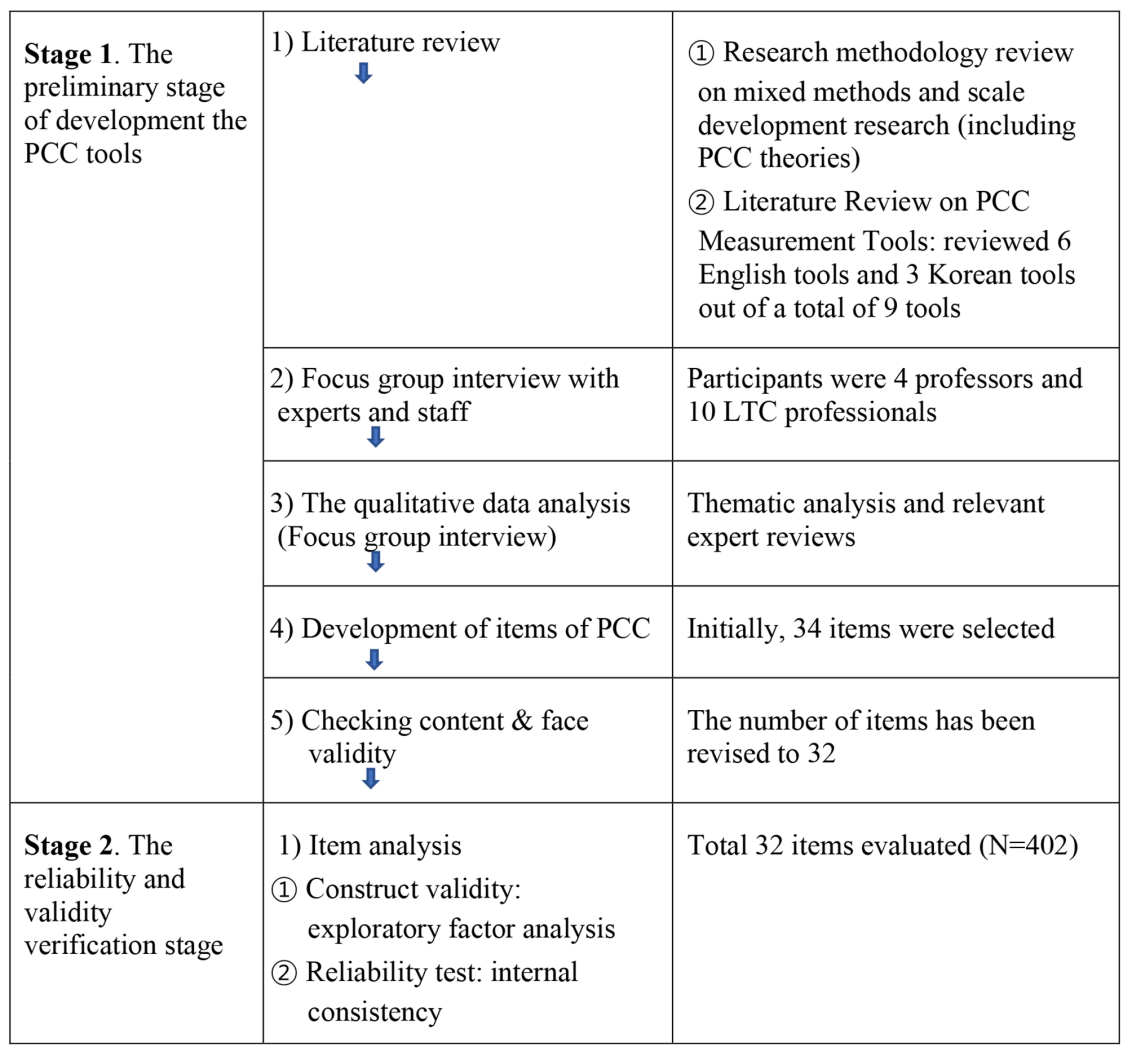
Phase of PCC-staff development

This is a methodological study to develop a PCC measurement tool that can be applied to the staff of residential facilities in South Korea. We used the mixed-method, quantitative and qualitative techniques for developing a more context-specific instrument by balancing two methodologies’ respective drawbacks [39]. This study consists of a tool development stage and reliability and validity verification stage for the tool [40]. In the tool development stage, tools were developed through a review of the existing literature, interviews with experts and staff related to residential facilities, and preliminary research. In the reliability and validity verification stage, 402 employees of residential facilities were investigated regarding a PCC tool, and the reliability and validity of this data was verified using a statistical method (Fig. 1).

### The preliminary stage of development the PCC tools

The theoretical framework for tool development was based on the properties analyzed using the mixed methods approach [41]. Mixed methods are widely practiced in the pre-tool development stage, and present empirical criteria through the theoretical stage, field stage, and final analysis stage, so this study proceeded as it is.

#### Literature reviews

At the theory review stage, focusing on “what is the core essence of PCC” and “what area and property the essence of concept is reflected and defined,” the domain and property of the concept and tentative definition were derived through systematic literature analysis. The scope of the literature review was searched for focused on studies with PCC attributes published in Korean and English in domestic and foreign academic journals, and the search terms “person,” “human,” and “resident” were “centered,” “focused,” and “directed” without year restrictions.

As a result of the extensive review of studies, the core domains of PCC could be summarized as follows: intimate relationships, self-determination, comfortable living environment for all residents, working environment and employee empowerment. If one item overlapped with the meaning of another item, only the more comprehensive item was chosen, and in the case of similar items, the clearer and more suitable item was selected. These areas are not independent of each other and influence each other in other areas, so that the more desirable one area is, the more desirable the other is; hence, more PCC is possible.

#### Focus group interview with experts and staff related to residential LTC facilities

To understand the PCC characteristics of LTC facilities, focus group interviews were conducted with 4 professors and 10 LTC professionals working in LTC service fields. The interview questions were as follows: “What do you think it means for residents to be person-centered in a residential facility?”; “How do you respect individual preferences and the individuality of residents?”; “What do you think is the most important aspect when assisting older adults in residential facilities?”; and “What kind of service would you like to receive if you later enter LTC facilities?” The interview was divided into two or three groups and lasted for one hour or up to one and a half hours per team.

#### The analysis of qualitative interview data

The contents of the research participants’ statements were recorded for the observation record, and the recorded interview data was documented as it is [42]. A theoretical record was made by extracting sentences or phrases meaning the PCC while repeatedly reading the documented recordings. The data were systematically recorded and organized so as not to deviate from the essence of the PCC while examining whether there were confirmed or omitted items. The in-depth interview data analysis used the thematic analysis method proposed by Waltz et al. [43] and was conducted simultaneously with data collection. Thematic analysis has the advantage of identifying recurring ideas and deriving a central theme. Meaningful contents were extracted, classified by case, and coded while assigning titles, and the list of categories was categorized into higher-level titles through coding. It was confirmed that the categories converged into the categories of intimate relationships, self-determination, home-like environment, and working environment, employee empowerment and organizational management identified in the literature review. As a result of the qualitative interview, it was possible to confirm the specific details of the items that were abstractly mentioned in the literature analysis. For example, “environment like home” means place residents with similar living habits in the same living space, “use wallpaper or hang pictures of the natural environment such as trees and flowers.”

In order to secure the validity of the analysis contents and results, one nursing professor with experience in LTC for older adults and qualitative research, and one nurse with more than 10 years of experience working in a nursing facility for older adults were reviewed and revised.

#### Development of items of PCC

The questionnaires to be included in the PCC area was arranged by analyzing the tools measuring PCC of previous studies. By comparing the interview contents with the areas and items arranged in the literature review, PCC measurement items that reflected the characteristics of LTC facility services were selected and adjusted.

The PCC tool developed in this study evaluates whether the staff perceive that the PCC is important, and whether the environment and system in the facility provide PCC services that both staff and participants feel PCC. Therefore, the area of ​​the question was divided into service environment and service provision. Service provision was further divided into intimate relationships, self-determination, and comfortable living environment for all residents, working environments and employee empowerment and organizational management. Through the abovementioned steps, the core domains of PCC could be summarized as follows: intimate relationships (4 items), self-determination (10 items), comfortable living environment for all residents (6 items), and working environment and employee empowerment (14 items). We selected 34 items that can measure the PCC of residential facilities. Since PCC service occurs based on the staff’s perception of PCC, therefore, this tool measures perceived PCC service.

In other words, the items evaluate the degree of PCC that staff themselves perceive about the institution’s environment and systems and whether they are providing PCC-like services. The overall degree of agreement was ascertained using a 6-point Likert scale with 1 = *strongly disagree* and 6 = *strongly agree*.

#### Verification of the tool through content validity and face validity

Content validity was conducted with 3 related researchers and 2 experienced personnel to find out whether the content and expression of the tool was appropriate. The two items were suggested to delete in the draft, where the degree of agreement among experts was less than a certain standard: “this area feels peaceful” and “if residents wish, they can live with their pets.” The preliminary survey was conducted in November 2019 with 32 questions targeting six experts related to older adult care services selected through convenience extraction. As a result of preliminary research, the overall degree of agreement was ascertained using a 6-point Likert scale with 1 = *strongly disagree* and 6 = *strongly agree*. The inter-rater agreement was high (66.7%~100.0%). The two items were deleted in the draft, where the degree of agreement among experts was less than a certain standard: “this area feels peaceful” and “if residents wish, they can live with their pets.” The draft tool was also revised by confirming ambiguity in expression or difficult items in content.

### The reliability and validity verification stage of the PCC tool

#### Survey participants

Research participants was the professionals working in residential LTC facilities nationwide through the cooperation of the Korea association of LTC centers for senior citizens. The sample size was finally determined by merging the empirical rule [44], which is known to be appropriate for a sample size of more than 200 in exploratory factor analysis, and the basis for calculating the sample size in confirmatory factor analysis [45]. With 374 degrees of freedom, a significance level of 0.05, and a test power of 95%, assuming that the model fit (RMSEA) of the null hypothesis is 0.00 and the alternative hypothesis is 0.05, the number of samples was calculated and 109 were calculated, and exploratory factor analysis and confirmation The number of participants sufficient to satisfy all of the positive factor analysis was set at 400, and the questionnaire was distributed to the final 405, considering the possibility of dropping out of about 1%. The survey participants agreed to the necessity of the study and responded to the questionnaire voluntarily, and the results of the questionnaire were returned by mail, e-mail, or online.

#### Survey process

Data collection was conducted from January 6 to January 31, 2020. Prior to data collection, approval was obtained from the Institutional Review Board (IRB). To increase the response rate and accuracy of responses, the purpose of the study, composition of the questionnaire, and response method were explained on the first page of the questionnaire.

After reading the research explanatory note and the questionnaire consisting of 34 items confirmed through preliminary, participants signed an intention to participate, and filled out a self-reported structured questionnaire for about 10 to 15 min. Of the returned 405 questionnaires, 3 had incomplete responses. Therefore, 402 cases were used in the study.

#### Data analysis

The collected data were analyzed using the SPSS 25 program. Descriptive analysis was performed on the general characteristics of the study participant and facilities to which the participant belonged, such as frequency, percentages, mean, and standard. Preliminary survey data confirmed the inter-rater agreement. The reliability and validity process used in this study was verified according to the principles presented in “guidelines for reliability, confirmatory and exploratory factor analysis” [46], and the proportion of variance was set at 5% or more according to the guidelines.

In this survey data, the question domain was classified through factor analysis. To verify the reliability and validity of the tool, Cronbach’s alpha, inter-domain correlations, and reliability coefficients of each domain, scores were analyzed according to survey participants by domain. By the domains of the tool, the correlation coefficient was computed by Spearman’s method. The difference in PCC scores by participant’s characteristics were calculated through the Kruskal-Wallis test with the post hoc comparison using Bonferroni’s correction.

#### Ethical considerations

Prior to the start of the research, ethics approval was obtained from the institutional ethics committee of the university to which the researcher belongs (1,041,493-A-2019-012). After explaining to all research participants about the purpose and content of the research, possibility of ending participation in the research at any time, and how the collected data will only be used for research purposes and confidentiality will be guaranteed, participants agreed to participate in the research and respond to the questionnaire with the participant’s signature.

## Results

### Participants’ and their affiliations’ general characteristics

Respondents were all women, and their average age was 57.82 ± 4.69 years old, 99.0% were caregivers, their work experience at the facilities was 4.73 ± 1.68 years. Married respondents constituted 85.57%, and high school graduates constituted 66.17%. In terms of self-evaluation, for “economic status” 43.78% selected “poor.” In the facilities where respondents worked, 93.78% were private facilities, and 30.35% of the facilities evaluated by the National Health Insurance Corporation were B-grade institutions. The average number of residents within the facilities was 34.71 ± 18.11 (Table 1).

**Table 1:** Characteristics of survey participants and their affiliation

Variable		Total(N = 402)
	40’s	27 (6.72%)
Age group (mean ± SD = 57.82 ± 4.69)	50’s	207 (51.49%)
	60’s	168 (41.79%)
Sex	Female	402 (100.00%)
Occupation	Caregiver	398 (99.00%)
	Social worker	1 (0.25%)
	Nursing assistant	3 (0.75%)
Work period (year)		4.73 ± 1.68
Work period group (year)	< 3	54 (13.43%)
	3 to < 5	152 (37.81%)
	≥ 5	196 (48.76%)
Marital status	Single	8 (1.99%)
	Married	344 (85.57%)
	Divorced	33 (8.21%)
	Widowed	17 (4.23%)
Educational level	Elementary school	1 (0.25%)
	Middle school	103 (25.62%)
	High school	266 (66.17%)
	College	31 (7.71%)
	University over	1 (0.25%)
Economic status	Very poor	-
	Poor	176 (43.78%)
	Normal	163 (40.55%)
	Enough	63 (15.67%)
	Very enough	-
	Private	377 (93.78%)
Operating administration, n (%)	Foundation	23 (5.72%)
	Social welfare corporation	1 (0.25%)
	No response	1 (0.25%)
Evaluation grade by NHIC, n (%)	A	17 (4.23%)
	B	122 (30.35%)
	C	82 (20.40%)
	D	8 (1.99%)
	E	-
Evaluation	No	173 (43.03%)
	Yes	229 (56.97%)
	Maximum number of patients	38.30 ± 19.13
Facility size	Current number of patients	34.71 ± 18.11
	Waiting number of patients	27.57 ± 15.09

### Construct validity of questionnaires

As a result of the factor analysis, 4 domains and 32 items concerning service conditions, resident’s right to self-determination, a comfortable living environment for all residents, and residents and staff satisfaction were derived, explaining 24.7%, 23.6%, 14.6% and 8.0% of the total variance, respectively (Table 2). “Service conditions” was a question that included a feeling of being comfortable and stable from the perspective of the residents, and the question as to whether “the staff here speak in a way that residents can understand” was the most influential. “Resident’s right to self-determination” is an area related to resident’s choice of preferences and needs. In particular, the question as to whether “the resident can change the caregiver if they wish to” was the most influential. Regarding “comfortable living environments for all residents” as an area related to an adequate working environment from an employee’s viewpoint, the question for “placing residents with similar lifestyles in the same living space” had the greatest influence. ”Resident and staff satisfaction” is a field related to workplace stability and satisfaction from an employee’s viewpoint, and the question where “employee satisfaction is high” had the greatest influence in the domain. The names of each factor were provided in consideration of the common meaning of the items included in the factors; when compared with the factor naming of other tools, they were semantically similar.

**Table 2:** Factor analysis of the items for the staff for checking the construct validity (N = 402)

Item	Factor1	Factor2	Factor3	Factor4
**Service Conditions**				
1. I feel welcome here.	**0.805**	-0.197	-0.288	0.226
2. I feel recognized as a person here.	**0.579**	-0.100	-0.277	0.494
3. Here I can be as I am.	**0.478**	-0.201	-0.384	0.607
4. Residents feel safe here.	**0.668**	-0.252	-0.407	0.093
5. The staff here speaks in a way that the residents can understand.	**0.816**	-0.184	-0.160	0.200
6. This place is homely.	**0.700**	-0.221	-0.381	0.172
7. This place has some nice things to see (e.g. landscapes, works of art, etc.).	**0.659**	-0.251	-0.520	0.285
8. I can’t have any unpleasant thoughts here.	**0.686**	-0.278	-0.444	0.284
9. This place is neat and clean.	**0.722**	-0.225	-0.474	0.260
10. Here it is easy for residents to keep in touch with loved ones (family, friends, etc.).	**0.656**	-0.227	-0.465	0.233
11. It is easy for residents to welcome visitors here.	**0.814**	-0.241	0.034	0.196
12. It is easy for residents to converse with staff here.	**0.721**	-0.360	-0.137	0.135
13. Here, the residents have someone they can talk to if they wish.	**0.728**	-0.149	0.048	0.198
**Resident’s right to self-determination**				
1. Residents can choose what to eat and when.	0.106	**-0.707**	-0.354	0.128
2. You can choose the time the resident wakes up.	0.388	**-0.704**	-0.087	0.190
3. Residents can choose whether or not to participate in the program and the type of program.	0.298	**-0.656**	-0.270	0.276
4. Residents can choose whether and when to bathe.	0.369	**-0.792**	-0.131	-0.020
5. The resident’s preferences are fully reflected in the care plan.	0.290	**-0.649**	-0.559	0.034
6. Residents can see a doctor whenever they want.	0.322	**-0.682**	-0.547	-0.024
7. Residents can honestly express their wishes to the staff at any time.	0.480	**-0.578**	-0.400	0.034
8. The resident can change the caregiver if they wish.	0.157	**-0.834**	-0.128	0.255
9. When a new employee arrives, the manager introduces them to the resident and encourages them to build close relationships.	-0.118	**-0.825**	-0.256	0.222
10. If the resident cannot make a decision on his/her own or if he/she does not understand it, discuss it thoroughly with his/her family.	0.268	**-0.815**	-0.021	-0.141
11. There is a space that can be utilized by reflecting the individual tastes and choices of residents.	0.350	**-0.687**	-0.173	0.370
12. If the resident wishes, there is a space where the family can come and stay.	0.215	**-0.637**	-0.421	0.068
**Comfortable living environment for all residents**				
1. Place residents with similar lifestyles in the same living space.	0.313	-0.416	**-0.524**	-0.164
2. Use wallpaper of natural environment pictures such as trees and flowers or hang a picture frame.	0.189	-0.530	**-0.735**	0.049
3. The atmosphere of the organization is equal and cooperative.	0.197	-0.320	**-0.726**	0.393
4. When establishing a care plan for residents, the opinions of direct service providers are fully reflected.	0.159	-0.226	**-0.656**	0.382
**Residents and staff satisfaction**				
1. If the resident wishes, he or she can grow flowers or vegetables.	0.390	-0.160	0.023	**0.446**
2. There are few employee turnover and there are many long-term employees.	0.209	-0.180	0.001	**0.470**
3. Employee satisfaction is high.	0.225	0.088	-0.228	**0.556**
Eigen value	7.911	7.554	4.681	2.588
Proportion of variance	0.247	0.236	0.146	0.081
Cumulative proportion of variance	0.247	0.483	0.63	0.710

Factors were derived from principal component analysis with oblimin rotation

These factors explained 71.0% of variance

### Internal consistency analysis of items

Cronbach’s alphas for service conditions, resident’s right to self-determination, a comfortable living environment for all residents, and residents and staff satisfaction for each domain were 0.965, 0.948, 0.652, and 0.525, respectively, thus demonstrating internal consistency (Table 3).

**Table 3:** Assessment results for each item using the questionnaire for LTC staff (N = 402)

Item	Mean if item excluded	SD if item exclusion	Modified item-total correlation coefficient	Square multiple correlation	Cronbach’s alpha if item excluded	Domain Cronbach’s alpha (95% asymptotic CI)
**Service conditions**						0.965 (0.960–0.970)
1. I feel welcome here.	32.242	19.564	0.674	0.984	0.961	
2. I feel recognized as a person here.	30.673	20.313	0.692	0.979	0.964	
3. Here I can be as I am.	33.060	19.820	0.691	0.979	0.964	
4. Residents feel safe here.	32.397	19.770	0.691	0.985	0.963	
5. The staff here speaks in a way that the residents can understand.	31.806	19.788	0.683	0.984	0.962	
6. This place is homely.	32.584	19.657	0.683	0.984	0.962	
7. This place has some nice things to see (e.g. landscapes, works of art, etc.).	32.490	19.555	0.676	0.983	0.961	
8. I can’t have any unpleasant thoughts here.	32.490	19.543	0.675	0.983	0.961	
9. This place is neat and clean.	32.407	19.513	0.672	0.982	0.960	
10. Here it is easy for residents to keep in touch with loved ones (family, friends, etc.).	32.314	19.661	0.683	0.982	0.962	
11. It is easy for residents to welcome visitors here.	30.051	20.330	0.692	0.986	0.964	
12. It is easy for residents to converse with staff here.	32.148	19.827	0.693	0.983	0.964	
13. Here, the residents have someone they can talk to if they wish.	31.340	20.298	0.708	0.986	0.966	
**Resident’s right to self-determination**						
1. Residents can choose what to eat and when.	38.461	18.468	0.638	0.969	0.944	0.948 (0.941–0.955)
2. You can choose the time the resident wakes up.	37.766	18.359	0.638	0.968	0.944	
3. Residents can choose whether or not to participate in the program and the type of program.	39.011	18.528	0.637	0.967	0.945	
4. Residents can choose whether and when to bathe.	38.348	18.332	0.631	0.967	0.943	
5. The resident’s preferences are fully reflected in the care plan.	40.836	17.686	0.625	0.964	0.940	
6. Residents can see a doctor whenever they want.	40.666	17.612	0.621	0.962	0.939	
7. Residents can honestly express their wishes to the staff at any time.	38.597	18.396	0.636	0.966	0.944	
8. The resident can change the caregiver if they wish.	38.695	18.196	0.630	0.966	0.943	
9. When a new employee arrives, the manager introduces them to the resident and encourages them to build close relationships.	40.451	17.863	0.638	0.965	0.944	
10. If the resident cannot make a decision on his/her own or if he/she does not understand it, discuss it thoroughly with his/her family.	40.010	18.007	0.644	0.965	0.946	
11. There is a space that can be utilized by reflecting the individual tastes and choices of residents.	37.466	17.233	0.636	0.968	0.946	
12. If the resident wishes, there is a space where the family can come and stay.	40.029	18.204	0.659	0.968	0.948	
**Comfortable living environment for all residents**						
1. Place residents with similar lifestyles in the same living space	29.176	16.749	0.287	0.480	0.555	0.652 (0.592–0.704)
2. Use wallpaper of natural environment pictures such as trees and flowers or hang a picture frame.	32.935	15.968	0.348	0.521	0.612	
3. The atmosphere of the organization is equal and cooperative.	29.397	15.435	0.262	0.435	0.500	
4. When establishing a care plan for residents, the opinions of direct service providers are fully reflected.	34.163	17.741	0.385	0.563	0.651	
**Residents and staff satisfaction**						
1. If the resident wishes, he or she can grow flowers or vegetables.	22.357	18.486	0.378	0.378	0.545	0.525 (0.439-0.600)
2. There are few employee turnover and there are many long-term employees.	34.639	19.290	0.264	0.264	0.418	
3. Employee satisfaction is high.	29.509	17.456	0.178	0.178	0.298	

### Inter-domain correlations and reliability coefficients

“The service conditions” was corelated with the resident’s right to self-determination (r = 0.643, p < 0.001), comfortable living environments for all residents (r = 0.674, p < 0.001), and the satisfaction of residents and staff (r = 0.526, p < 0.001). There was also a strong positive correlation between resident’s right to self-determination and comfortable living environments for all residents (r = 0.695, p < 0.001). Although there was no significant positive correlation between the resident’s right to self-determination and resident and staff satisfaction, or between comfortable living environments for all residents and resident and staff satisfaction, it can be interpreted that convergence validity was secured (Table 4).

**Table 4:** Inter-domain correlations and reliability coefficients of each domain

Variable	No. of items	V1	V2		V3		V4	
Service condition (V1)	13	1						
Resident’s right to self-decision (V2)	12	0.643 (p < 0.001)	1					
Comfortable living environment (V3)	4	0.674 (p < 0.001)	0.695 (p < 0.001)		1			
Satisfaction of residents and staff (V4)	3	0.526 (p < 0.001)	0.179 (p < 0.001)		0.290 (p < 0.001)		1	

Correlation coefficient was computed by Spearman’s method

### PCC scores of caregivers by their characteristics

The PCC score (with a total of 192 points) was compared to the evaluation results of residential LTC facility to which the respondent belongs and the respondent’s characteristics. The tool’s validity was confirmed by responding that the PCC of the affiliated facility was significantly higher, as the number of personnel working in a facility and A-grade evaluation result of residential LTC facility. According to the characteristics of the survey participants and their affiliations, being older, having more than 3 years of experience, a higher educational level, or higher economic level indicated a higher perceived PCC level (Table 5).

**Table 5:** PCC Scores according to survey participants and their affiliation (N = 402)

	Variable	N	Total		
			Mean ± SD		p-value
Evaluation grade of facilities, n (%)	A (Reference)	17	161.18 ± 6.67		< 0.001
	B	122	133.66 ± 10.04***		
	C	82	123.96 ± 5.23***		
	D	8	112.12 ± 0.35***		
	No evaluation	173	141.06 ± 12.09***		
Age group	40’s (Reference)	27	127.56 ± 11.59		0.003
	50’s	207	135.70 ± 14.26**		
	60’s	168	136.79 ± 12.27***		
Work period group (year)	< 3 (Reference)	54	133.13 ± 13.30		0.258
	3 to < 5	152	136.10 ± 13.17		
	≥ 5	196	135.90 ± 13.70		
Marital status	Single (Reference)	8	130.00 ± 0.00		0.137
	Married	344	136.15 ± 13.52		
	Divorced / widowed / others	50	132.74 ± 13.59		
Educational level	< High school (Reference)	104	131.35 ± 9.70		0.001
	High school	266	136.77 ± 13.06***		
	> High school	32	139.72 ± 21.89		
Economic status	Poor (Reference)	176	129.66 ± 10.31		< 0.001
	Normal	163	135.37 ± 13.14***		
	Enough	63	152.81 ± 4.15***		

P-value was calculated by Kruskal-Wallis test with the posthoc comparison using Bonferroni’s correction

*, p < 0.05; **, p < 0.01; ***, p < 0.001 compared with reference

The PCC is a total of 192 points, 32 items x 6-point Likert scale per item, from 1 = *strongly disagree* to 6 = *strongly agree*

## Discussion

We developed a PCC measurement to evaluate the perception of staff working in LTC facilities and whether they consider if PCC is important for older adults. We examined if staff perceived that the environment and system in the facility provide PCC services using 4 domains and 32 items, concerning service conditions, resident’s right to self-determination, a comfortable living environment for all residents, and residents and staff satisfaction. These were identified as the PCC-staff version of residential LTC facilities.

As a result of factor analysis, the construct validity and the internal consistency of this tool is high. Using this tool, caregivers will try to create PCC conditions and provide services, and recipients will be able to know PCC services and improve the quality of life in the facility. In other words, the PCC service can be provided to care recipients only when the staff themselves acknowledge the importance of PCC for the participant and the environment and system in the facility are equipped to PCC to both the employee and the participantItems in other domains were also from this point of view, and the PCC evaluation tool can be a useful tool that improves the quality of life of the older residents and affects the job satisfaction and turnover rate of care workers.

The person-centered climate questionnaire-staff version developed by Edvardsson et al., which has been widely used in many studies by translating it into the native language, is a total of 13 questions in 3 categories: 7 items of personalizing, 4 items of organizational support, and 2 items of environmental accessibility [32]. Service conditions in our study is similar to environmental accessibility, and resident’s right to self-determination can be matched to personalizing, while a comfortable living environment for all residents and resident and staff satisfaction consists of similar questions to organizational support. However, as measurement items were added through the on-site interviews, the number of items has increased.

In a previous study, PCC was analyzed to have a significant effect on the reduction of behavioral and psychological symptoms, such as depression and nervousness, and a meaningful increase in social activities, as well as an improvement in quality of life in older adult with dementia [47]. In a review study, it was confirmed that applying PCC in residential care facilities helps residents cope with smoking and drinking problems [48]. It has been confirmed that forming a close relationship with residents based on human-centered care can have a positive effect on job stress reduction and job satisfaction even though personnel who care for older adults with dementia tend to have low job satisfaction and are prone to exhaustion, due to the problem behaviors and cognitive symptoms of older adults [49].

In South Korea, the PCC Assessment Questionnaire for Daycare Center Staff consisted of a total of 20 questions in 3 areas, as follows: Intimate relationships and environment (10 items), consumers’ self-determination (6 items), and home-likeness (4 items) [34]. The service of daycare centers are an intermediate type between residential facilities and home services, and it is difficult to compare them precisely. However, the similarity with the areas and items of the tools developed in our study was high. Thus, the service conditions derived in this study is the home-likeness of the PCC assessment tool for daycare centers, and the resident’s right to self-determination is the consumers’ self-determination. Intimate relationships and the environment can be understood as similar to a comfortable living environment for all residents, and the satisfaction of residents and staff. However, the first Swedish version of the person-centered climate questionnaire-resident version in 2008 consisted of three items: “safety,” “generosity,” and “routineness” [33]. In the study of Yoon et al. in South Korea [26], 17 items were classified into two factors (i.e., everydayness, safety). There was a difference in emphasizing the safety of the service, daily comfort, and generous attitude of the institution compared to the staff version.

The PCC score (192 points) was compared according to the evaluation result of the residential care facility to which the respondent belongs and the characteristics of the respondent (Table 4). The tool’s validity was confirmed by responding that the PCC of the affiliated facility was significantly higher, as the number of personnel working in a facility with an A-grade evaluation result of residential care facility. If the basic human resources training system is in operation and employees have a high awareness of the PCC and a will to practice it.

According to the characteristics of the employees, the higher the age, an additional three years of work experience is added, the higher the educational level, the higher the level of the economic status, and the higher the recognition of the PCC level, the more likely it is for employees to offer a PCC. This can be interpreted as a high probability of providing PCC when employees enjoy the LTC job for more than simply making money; there is no financial difficulty, and they enjoy the job enough to work for 3 years or more. This may demonstrate the validity of this tool more accurately.

To implement PCC in the previous study, it was emphasized that it is important to transparently manage daily records and create conditions where employees can respond to the needs of residents, not only in the records but also in actual practices [50]. This shows that caregivers can provide LTC services while receiving regular education and training and acquire various experiences in the process of providing services, so that relational and emotional aspects can work during the service process. LTC institutions should support their staff in making these efforts [50].

This study had the following limitations and suggests future research directions as follows. Firstly, older people residing in facilities were not included in the interview for tool development, even though they were receiving services, because there were many older people suffering from dementia and unable to respond [51]. However, we believe that these limitations have been some extent supplemented through literature reviews. Besides, the reliability and validity of self-ratings is questionable in the later stages of dementia, and the use of proxy measures is preferred in advanced dementia and for longitudinal evaluations [52]. The proxy‐assessment is typically performed by family members or caregivers in a close relationship to the person with dementia [53, 54]. In this respect, it is judged that interviews and surveys through caregivers as their proxy would have supplemented the reality of the tool to some extent. Secondly, this tool cannot measure how much PCC employees actually provide; it uses only the self-rating of employees, as with other studies. Numerous studies have used the self-rating method as a surrogate variable for actual behavior, but there is a limitation for not confirming the relationship between the two [55]. Future research should verify this and adjust the PCC measurement tool to measure actual care behavior. In addition, it is necessary to continuously evaluate the effect of the degree of PCC on the physical health level of the participant, psychological stability such as depression and anxiety, and quality of life. Thirdly, in the future, studies that repeatedly analyze the validity and objectivity of this tool in various settings are needed.

## Conclusions

PCC is a philosophical and practical agenda that is considered important to the overall healthcare system and especially in the field of LTC for older adults. Developing a PCC model for LTC facilities in South Korea, improving the quality of services by using the tools [26] that measure the perception of residents, and developing tools [25, 56] centered on staff may be useful for this purpose. In this study, a tool was developed to evaluate how person-centered services that are provided in residential care facilities are based on the views of staff, and reliability and validity of the tool was verified. A tool that consisted of 32 questions in a total of 4 areas was derived. It was confirmed that reliability and validity above a certain standard were secured.

## Data Availability

The datasets are available from the corresponding author through the meeting of the Ethics Committee.

## List of abbreviations

NHIC: National Health Insurance Corporation
LTC: Long-term care
PCC: Person-centered care
PCECAT: Person-Centered Environment and Care Assessment Tool
